# Case‐fatality and disability in the Tanzanian Stroke Incidence Project cohort

**DOI:** 10.1111/ane.12422

**Published:** 2015-05-05

**Authors:** R. W. Walker, K. Wakefield, W. K. Gray, A. Jusabani, M. Swai, F. Mugusi

**Affiliations:** ^1^ Northumbria Healthcare NHS Foundation TrustNorth Tyneside General HospitalNorth ShieldsTyne and WearUK; ^2^ Institute of Health and SocietyNewcastle University, Newcastle‐upon‐TyneUK; ^3^ Kilimanjaro Christian Medical CentreMoshiTanzania; ^4^ Department of MedicineMuhimbili University College HospitalDar‐es‐SalaamTanzania

**Keywords:** stroke, Tanzania, case‐fatality, mortality, sub‐Saharan Africa, disability

## Abstract

**Objectives:**

The burden of stroke on healthcare services in sub‐Saharan Africa (SSA) is increasing. However, long‐term outcomes from stroke in SSA are not well described. We aimed to investigate case‐fatality and health outcomes for stroke survivors at 7‐ to 10‐year follow‐up.

**Materials and methods:**

The Tanzanian Stroke Incidence Project (TSIP) recruited incidence stroke cases between 2003 and 2006. We followed up cases in 2013, recording date of death in those who had died.

**Results:**

Of 130 stroke cases included in this study, case‐fatality and date of death data were available for 124 at 7–10 years post‐stroke. Of these, 102 (82.3%) had died by 7 years post‐stroke. Functional disability, as measured by the Barthel index immediately post‐stroke, was a significant predictor of case‐fatality at seven‐year follow‐up with those with severe disability having an almost four‐fold increase in the odds of death compared with those with no, mild or moderate disability.

**Conclusions:**

Case‐fatality rates are higher than reported in high‐income countries, with post‐stroke disability a significant predictor of death. Sustainable interventions to reduce post‐stroke disability in this setting should be investigated.

## Introduction

The burden of stroke in sub‐Saharan Africa (SSA) appears to be increasing, and post‐stroke disability rates in some areas are as high as in high‐income countries 1, 2. Although the reasons for this are likely to be multifactorial, demographic ageing and a reducing burden of communicable diseases, such as malaria, tuberculosis and HIV/AIDS, may be key factors.

Post‐stroke outcomes vary widely between, and within, world regions depending on a range of factors including demographic profile, stroke type and severity and immediate and long‐term post‐stroke care 3, 4, 5, 6. However, long‐term post‐stroke outcomes in SSA are poorly described, with most studies being hospital‐based. As the majority of stroke cases may not attend hospital, such data cannot be relied upon to give an accurate picture of the epidemiology of stroke in SSA 7. Furthermore, few studies look at outcomes beyond 30 days post‐stroke. Nevertheless, there have been a few recent studies, which have added to our knowledge of post‐stroke outcomes in SSA. A study of 651 hospitalized stroke cases in Maputo, Mozambique, found that initial stroke severity and in‐hospital complications were the main determinants of 28‐day case‐fatality 8. A study from Johannesburg, South Africa, followed up 200 hospitalized first‐time ischaemic strokes and found 38% case‐fatality at 12 months 9. Mortality was higher in those with poorest functional ability, as measured by the Barthel Index, at discharge. In this resource‐poor setting, the average length of hospital stay was only 6 days and so functional ability is likely to be a strong marker for stroke severity, thus supporting the findings from Maputo. Danesi and colleagues reported 30‐day case‐fatality of 16.2% in a community‐based study of 160 stroke cases in Lagos‐state, Nigeria 10. However, all cases had to present at a health facility and prehospitalization deaths were not included, meaning that not all cases will have been included. Finally, a study of 272 hospital admissions from Abuja, Nigeria, reported 30‐day case‐fatality rates of 18.8% 11.

We have previously reported 28‐day (23.8%) and 3‐year (60.0%) case‐fatality rates for 130 cases recruited to the Tanzanian Stroke Incidence Project (TSIP) between 2003 and 2006 12. TSIP was a community‐based stroke incidence study. We have also published details of correlates of 28‐day and 3‐year case‐fatality 6. At both time points, markers for stroke severity (functional ability, speech, language, swallowing, confusion, hearing and gaze paresis) were the main correlates.

The main aim of this study was to investigate outcomes for TSIP survivors at 7–10 years post‐stroke. Key outcomes of interest were case‐fatality and disability levels.

## Methods

### Ethics

Ethical approval for this study was obtained from the National Institute of Medical Research in Tanzania and from the Newcastle and North Tyneside Joint Ethics Committee. Consent was sought for baseline data collection. For those who were unable to consent due to physical or cognitive impairment, assent was obtained from a close relative.

### Participants and setting

Patients were recruited from survivors of those identified by the TSIP, which recruited incident cases of stroke occurring between June 2003 and June 2006 in the rural Hai district and urban Dar‐es‐Salaam, Tanzania 7. Only those who met the World Health Organization criteria for stroke were included 13. Stroke patients were not followed up in Dar‐es‐Salaam. The Hai district is a rural area in northern Tanzania, and part of it has been used as a demographic surveillance site (DSS) since the 1990s. The population of the DSS at the mid‐point of recruitment was estimated to be 159,814 living in 52 villages. Subsistence farming is the main source of income for the vast majority of the population, although some wealthier families are able to sell cash crops, such as coffee, for additional income.

The TSIP used two methods of case ascertainment. Firstly, prospective case identification of those living within the community, or admitted to health facilities, who survived long enough to undergo assessment. Secondly, identification of those who died rapidly after stroke using a system of verbal autopsy set‐up to record information regarding cause of death for all people dying within the DSS during the study period 14. Within the Hai DSS, the age‐adjusted yearly incidence of stroke was 108.6 per 100,000 (95% CI 89.0–130.9). Full details of the study methods have been published previously 7. Mortality data for those identified by verbal autopsy have already been published 12. The data presented here relate to cases identified by prospective case identification. We identified 132 cases by this method. However, one case of subarachnoid haemorrhage was excluded from further analysis due to the different aetiology of the condition, and for one case, we did not have a date of death, giving a potential follow‐up cohort of 130.

### Follow‐up in 2013

Survivors of the TSIP cohort in Hai have been followed up previously in 2008 15. In the current study, survivors were followed up in June–July 2013 (7–10 years post‐stroke).

### Data collection

At baseline, demographic and post‐stroke function data were collected. Data were collected relating to functional ability in activities of daily living (Barthel Index) 16. Severe disability in activities of daily living was defined as a score of 0‐14, moderate disability as 15–18 and mild or no disability as 19–20 17. In patients still alive, computerized tomography (CT) head scans were recorded where the patient was able and willing to travel to hospital to undergo scanning. CT scans were analysed independently by a general radiologist at Kilimanjaro Christian Medical Centre, Tanzania (A.J.), and a neuroradiologist from Newcastle General Hospital, UK, and the diagnosis of cerebral infarct or haemorrhage compared and a consensus diagnosis reached 18. Intracerebral bleeds were classified as haemorrhagic strokes. Findings of ischaemia, haemorrhagic infarct or no evidence of stroke were classified as infarction. At follow‐up, data on case‐fatality and date of death were recorded.

### Statistical methods

Data were analysed using standard statistical software, *SPSS‐18 for windows* (SPSS, Chicago, IL, USA). Confidence intervals (CIs) for percentages are based on the normal approximation to the binomial distribution. A lack of overlap in CIs when comparing males and females indicates statistical significance. Correlation was assessed using the point biserial method.

We used survival analysis to investigate our data. Survival analysis is designed to adjust for the fact that some individuals are recruited to a study earlier or later than other. In general, at a given follow‐up point, those recruited earlier will have had a greater chance of a critical event (e.g. death) occurring, because they have been in the study for longer. In our study, those cases who died prior to follow‐up had reached the critical end point and those still alive at the end of the study period were described as censored. A Kaplan–Meier survival curve is presented. Cox proportional hazards regression modelling was used to investigate the influence of disability on case‐fatality. The model was adjusted for the possible effects of age at stroke and gender on the outcome. The outputs from the models are expressed in terms of the hazard ratio.

To simplify the model interpretation, for the purposes of the Cox regression model, age at stroke data were used untransformed and uncategorized and Barthel index scores were dichotomized into severe disability (score 0–14) and no, moderate or mild disability (score 15–20).

## Results

**Figure 1:**
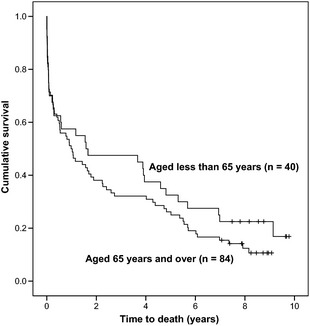
Kaplan–Meier survival curve.

Of the follow‐up cohort of 130 cases, six cases, known to be alive in 2009, could not be traced in 2013 and they were excluded. Of the remaining 124 cases, 102 (82.3%) had died by 7‐year follow‐up. Case‐fatality rates in yearly intervals from 3 to 7 years post‐stroke are presented in Table 1, and a Kaplan–Meier survival curve is presented in Fig. 1, with cases split into those aged 65 and over (*n *=* *84), and those aged less than 65 (*n *=* *40), at the time of the incident stroke. As shown in Table 1, there was very little difference in survival rates between males and females. At 7 years post‐stroke, there was no significant difference in ages between those who died and those who survived (*t* = 1.854, *P *=* *0.066, 122 degrees of freedom). The mean age at death was 71.9 years (standard deviation 13.40).

**Table 1:** Case‐fatality rates at 3–7 years

Years since stroke	3 years	4 years	5 years	6 years	7 years
Total number of deaths (number/sample size (percentage, 95% CI))	78/130 (60.0%, 95% CI 51.6–68.4)	82/124 (66.1%, 95% CI 57.8–74.5)	89/124 (71.8%, 95% CI 63.9–79.7)	97/124 (78.2%, 95% CI 71.0–85.5)	102/124 (82.3%, 95% CI 75.5–89.0)
Number of male deaths (number/sample size (percentage, 95% CI))	42/69 (60.9%, 95% CI 49.4–72.4)	45/64 (70.3%, 95% CI 59.1–81.5)	47/64 (73.4%, 95% CI 62.6–84.3)	51/64 (79.7%, 95% CI 69.8–89.5)	53/64 (82.8%, 95% CI 73.6–92.1)
Number of female deaths (number/sample size (percentage, 95% CI))	36/61 (59.0%, 95% CI 46.7–71.4)	37/60 (61.7%, 95% CI 49.4–74.0)	42/60 (70.0%, 95% CI 58.4–81.6)	46/60 (76.7%, 95% CI 66.0–87.4)	49/60 (81.7%, 95% CI 71.9–91.5)
Mean age of those who died (standard deviation)	71.2 (13.97)	70.3 (14.32)	70.4 (14.07)	70.4 (13.69)	70.2 (13.44)
Mean age of those alive (standard deviation)	65.7 (15.06)	66.8 (14.96)	65.9 (15.53)	64.4 (16.81)	64.0 (18.46)
Number of deaths in those with no/mild disability (number/sample size (percentage, 95% CI))	5/16 (31.3%, 95% CI 8.5–54.0)	5/15 (33.3%, 95% CI 9.5–57.2)	5/15 (33.3%, 95% CI 9.5–57.2)	5/15 (33.3%, 95% CI 9.5–57.2)	5/15 (33.3%, 95% CI 9.5–57.2)
Number of deaths in those with moderate disability (number/sample size (percentage, 95% CI))	3/9 (33.3%, 95% CI 2.5–64.1)	3/8 (37.5%, 95% CI 4.0–71.0)	3/8 (37.5%, 95% CI 4.0–71.0)	3/8 (37.5%, 95% CI 4.0–71.0)	4/8 (50.0%, 95% CI 15.4–84.6)
Number of deaths in those with severe disability (number/sample size (percentage, 95% CI))	42/75 (56.0%, 44.8, 67.2)	45/72 (62.5%, 95% CI 51.3–73.7)	52/72 (72.2%, 95% CI 61.9–82.6)	60/72 (83.3%, 95% CI 74.7–91.9)	64/72 (88.9%, 95% CI 81.6–96.1)

CI, confidence interval.

### Stroke subtype and case‐fatality

CT head scan data, performed within 15 days of stroke, was available for 57 of the 124 cases followed up (46.0%). Of these, 48 (84.2%) had evidence of a stroke caused by cerebral infarct and 9 (15.8%) had evidence of a stroke caused by a haemorrhage. At 7 years post‐stroke, only one case (11.1%) with evidence of a haemorrhagic stroke was still alive compared with 10 cases (20.8%) still alive in those who had had a cerebral infarct. This difference was not significant (*χ* ^2^ (1) = 0.460, *P *=* *0.498).

### Stroke severity and case‐fatality

The Barthel index score at first post‐stroke assessment was used as an overall marker for stroke severity. At 3 years post‐stroke, baseline Barthel Index data were available for 100 cases (50 alive), and at 7 years post‐stroke, baseline Barthel Index data were available for 95 cases (22 alive). Mortality data split into disability level are presented in Table 1. Of those still alive at 7 years, 10 (45.5%) had mild/no disability, 4 (18.2%) had moderate disability, and 8 (36.4%) had severe disability. In comparison, for those who had died, 5 (6.8%) had no/mild disability, 4 (5.5%) had moderate disability, and 64 (87.7%) had severe disability at baseline. As at 28 days (*r* = 0.277, *P *=* *0.005) and 3 years (*r* = 0.330, *P *=* *0.001) post‐stroke 12, disability in functional activities of daily living post‐stroke was strongly correlated with case‐fatality at 7 years (*r* = 0.522, *P *<* *0.001).

At seven‐year follow‐up, Cox regression revealed having severe disability to be a significant predictor of case‐fatality, even after adjusting for the effects of age at stroke and gender (hazard ratio = 3.906, 95% CI 1.844–8.272, *P *<* *0.001). Interestingly, neither age at stroke or gender was a significant predictor of case‐fatality.

Considering only those who were still alive in June 2009, and for whom follow‐up data were available (*n *=* *45), Barthel index score immediately post‐stroke was still a significant correlate of case‐fatality at 7 years post‐stroke (*r* = 0.610, *P *<* *0.001). For 23 people who were dead at 7‐year follow‐up, 22 (95.7%) had severe disability and 1 (4.3%) had moderate disability. In contrast, of the 22 still alive, 8 (36.4%) had severe disability, 4 (18.2%) had moderate disability, and 10 (45.5%) had mild or no disability.

## Discussion

This is the first long‐term follow‐up of incident stroke cases conducted in SSA. At seven‐year follow‐up, over 80% of cases had died. It may be argued that as the length of time since stroke increases, then the survivors are likely to be those whose functional ability was least affected. This appears to be the case for our cohort, with 88.9% of those with severe post‐stroke disability dead at 7 years, compared with 39.1% of those with no, mild or moderate disability. In regression modelling, initial post‐stroke disability appears to be a more important predictor of case‐fatality at 7 years than age and gender. The high prevalence of functional disability in stroke survivors living in Tanzania has been noted previously 19.

As shown in Fig. 1, those who were older at the time of stroke had consistently higher risk of mortality than those who were younger. Nevertheless, the mean age at stroke of those who died has changed little from 3‐year to 7‐year follow‐up, 71.2 and 70.2 years, respectively, emphasizing the lack of association between age and case‐fatality 12.

Our findings in relation to the role of immediate post‐stroke disability in predicting mortality support those of Mudzi et al. 9 who studied 200 first‐time ischaemic stroke admissions to a teaching hospital in Johannesburg, South Africa. Seventy‐six patients (38%) had died at 12‐month follow‐up. Barthel index score, Rivermead Mobility Index score 20 and health‐related quality of life (EuroQOL‐5D 21) were all recorded on admission to hospital, and all were significantly associated with 12‐month case‐fatality, although age was not. Mean Barthel Index score was 6.6 (95% CI 6.2–7.0) in survivors and 5.2 (95% CI 4.7–5.7) in those who died (*P *<* *0.001). At 5 years of our Tanzanian cohort, case‐fatality rates (71.8%) were noticeably higher than reported in Denmark (60%), Australia (58%) and the UK (53%) 22, 23, 24. Higher survival rates in these high‐income countries may be, in part, a reflection of the higher levels of long‐term care and support available. Although stroke subtype was not a significant predictor of case‐fatality, the small number of cases involved is likely to have resulted in a type II statistical error.

We are unable to comment on the exact cause of death in our cohort. Such data are not collected routinely in this setting. However, in the absence of community‐based medical care, post‐stroke complications such as pressure sores and increased risk of respiratory infections, are likely to be common. Mental health issues related to social isolation, such as depression, are likely to exacerbate the situation.

The main limitation of our study is that our results are based on those patients that survived long enough to be identified, interviewed and assessed by the TSIP, although time‐to‐death data for those who were identified by verbal autopsy have been reported previously 12. Data relating to disability levels in those identified by verbal autopsy may have altered our conclusions. Even in the 130 cases recruited to the TSIP, the 95 who had Barthel Index data available were those TSIP cases who survived long enough to be assessed, with those who were not assessed likely to have had more severe strokes. However, collecting data on post‐stroke functional ability (or stroke severity) for those dying rapidly after stroke would be challenging in any setting, and even a large‐scale prospective cohort study may not yield such data. We recognize that the sample size in relation to functional outcomes from 3 years post‐stroke is small, and this limits the generalizability of our findings and the data should be interpreted with caution. Nevertheless, given the lack of previous data, we feel our results add substantially to current knowledge. We are unable to present reliable data on cause of death in our cohort. Such data are not routinely recorded in Hai. Although verbal autopsy has been used by our team to identify stroke as a cause of death, it is not always reliable for other causes of death. Finally, our manuscript focussed on the role of post‐stroke disability and demographic factors on survival. The number of survivors at 7 years was too small to allow meaningful analysis of a wider range of variables as predictors of case‐fatality. However, correlates of three‐year case‐fatality in this cohort have been described previously 6.

In summary, case‐fatality rates at 7–10 years post‐stroke are higher than those reported in high‐income countries, with severe disability a significant predictor of case‐fatality. Sustainable interventions to reduce post‐stroke disability in this setting should be investigated.

## Conflict of interests

There were no conflicts of interests.

## Sources of Funding

The Tanzanian stroke incidence study was funded by a grant from the Wellcome Trust, UK (grant number 066939).

## Author Contributions

RW, KW and WKG designed this follow‐up study. MS, AJ and FM organized baseline data collection. KW collected and collated the follow‐up data. WKG did the literature review, the data analysis and wrote the first draft of the manuscript. All authors critically reviewed and revised the final manuscript.
